# Paving the road to recovery: the colorectal surgery ERAS pathway during the COVID-19 pandemic

**DOI:** 10.1093/bjs/znab208

**Published:** 2021-07-06

**Authors:** C J G Goodmaker, M Kopczynska, R Meskell, D Slade

**Affiliations:** Department of Colorectal Surgery, Salford Royal Hospital, Stott Lane, Salford, M6 8HD, UK

*Dear Editor*,

NHS England reports that 4.7 million people are awaiting an operation in England, the highest in a decade. Healthcare systems are seeking methods to limit an accumulating impact on services. Clinical pathways can be used to improve patient outcomes when recovery is relatively predictable^1^. The Enhanced Recovery After Surgery (ERAS) pathway has been shown to reduce rates of postoperative complications, reduce length of stay, and readmission and mortality rates^2^. Implementation of this pathway during and after the COVID-19 pandemic could help reduce time spent in hospital, exposure to nosocomial infections, improve patient flow and help clear waiting lists. Preliminary data from Italy, however, suggested that a colorectal surgery ERAS pathway could not be implemented effectively during a national crisis^3^. Conversely, although supporting data are limited, it has been speculated that ERAS could play a vital role in the UK’s response to the pandemic^4^.

In conjunction with national guidance^5^ the authors’ tertiary colorectal centre implemented a brief moratorium on elective procedures during March and April 2020. Urgent and emergency cases received rapid SARS-CoV-2 PCR swab testing. Beyond April, elective cases were reintroduced with a pre-admission 14-day isolation period and 3-day preoperative SARS-CoV-2 PCR swab.

The effect of the COVID-19 pandemic on colorectal ERAS pathway adherence and patient outcomes was explored. Complete methodology and demographics can be found in *Table* *S1*. Patients were split into two cohorts; those admitted before (January 2019—February 2020, 110 patients) and during (March 2020—December 2020, 56 patients) the pandemic (*Table 1*). Compliance with 14 ERAS pathway factors, spanning 3 days after the operation and shown to be influential on postoperative outcomes^2^, was assessed retrospectively (*Table* *S**2*).

**Table 1:** Surgical pathway adherence represented by compliance to the ERAS pathway and surgical approach and postoperative outcomes for colorectal ERAS patients before and during the pandemic

	**Before pandemic** **(*n* = 110)**	**During pandemic** **(*n* = 56)**	**Overall** **(*n* = 166)**	***P*** ^†^
**Pathway adherence**				
**Mean ERAS compliance (%)**	58.6	68.2	61.8	<0.001
**Surgical approach**				
Laparoscopic	41 (37.3)	27 (48.2)	68 (41.0)	0.175
Planned open	52 (47.3)	22 (39.2)	74 (44.6)	0.328
Converted to open	17 (29.3)	4 (19.9)	21 (23.6)	0.082
Robotic	0 (0)	3 (5.4)	3 (1.8)	–
**Postoperative outcomes**				
Length of stay (days)^*^	7 (5–12)	7 (4–10)	7 (4.75–11)	0.172
Complications	55 (50.0)	23 (41.1)	78 (47.0)	0.276^‡^
30-day post-discharge readmissions	10 (9.1)	3 (5.4)	13 (7.8)	0.397^‡^
30-day post discharge mortality rate	0 (0)	0 (0)	0 (0)	N/A

Values in parentheses are percentages unless indicated otherwise;

*values are median (i.q.r.).

^†^Mann–Whitney *U* test, except.

^‡^χ^2^ test.

Mann–Whitney *U* test analysis revealed an increase in colorectal surgery ERAS pathway compliance during the pandemic (*P* < 0.001) compared with before (*Table 1* and *Fig*. *S1*). No significant difference in length of stay (*Table 1* and *Fig*. *S**2*) was observed. This was on the background of a reduction in admissions to 80 during year 2020 from 104 (median over past 10 years). Analysis using χ^2^ test did not reveal a significant difference in postoperative complications (*Table**S**3*) or 30-day post-discharge readmission rates (*Table 1*). The 30-day post-discharge mortality rate remained at zero in both groups and no patients tested positive for SARS-CoV-2 PCR swabs during admission.

The colorectal surgery ERAS pathway has passed the ultimate stress test; it is safe, straightforward and improves outcomes. It should form part of the post-pandemic recovery with the aim to clear waiting lists.

*Disclosure*. The authors declare no conflicts of interest.

## Supplementary material

Supplementary material is available at *BJS* online.

## Supplementary Material

znab208_Supplementary_Data
