# Supplementary material for: Paving the road to recovery: the colorectal surgery ERAS pathway during the COVID-19 pandemic
Source: Br J Surg. 2021 Jul 6:znab208. doi: 10.1093/bjs/znab208 (PMC8406880; doi:10.1093/bjs/znab208)

**Appendix**

**Methods**

A database of 182 patients who underwent elective colorectal operations at Salford Royal Hospital (January 2019 - December 2020) and followed the colorectal surgery Enhanced Recovery After Surgery (ERAS ) pathway were included in the study. Of these 16 were excluded due to lack of data in the electronic patient records system. Patient demographics are presented in *Supplementary Table 1*. Compliance with 14 instrumental ERAS factors was assessed retrospectively (*Supplementary Table 2*). Compliance was calculated as a percentage of factors confirmed to have been completed by cross referencing clinical notes, drug charts and laboratory reports on the electronic patient records system. The surgical approach, length of stay, complications (*Supplementary Table 3*), re-admission and mortality rates were prospectively collected in the original database. Statistical analysis was performed using SPSS Version 26. Mann-Whitney U test was used to measure the effect of the pandemic on mean ERAS pathway compliance and length of stay. Chi squared test was used to assess the effect of the pandemic on individual ERAS factor compliance, complications, operative approach and re-admission rates.

**Patient demographics**

Of 166 patients included in the analysis, 53.6% were male and 46.6% female. The median age was 66 years (IQR 55 – 73.25). The majority of patients were scored American Society of Anaesthetists (ASA) grade 2 – 62.7%%. The most common operation was a Right Hemicolectomy (29.5%) followed by Anterior resection (20.5%). The main indication for operation was for the management of colorectal cancer (75.9%) followed by ulcerative colitis (9.0%). The majority of these operation were performed laparoscopically (44.6%) with 41.0% performed openly and 12.7% converted to open.

| **Patient characteristics** | **All patients (n = 166)** |
| --- | --- |
| Age - median, IQR | 66 (55 – 73.25) |
| ***Sex*** | |
| Male | 89 (53.6%) |
| Female | 77 (46.4%) |
| ***ASA grade - no. (%)*** | |
| 1 | 6 (3.6%) |
| 2 | 104 (62.7%) |
| 3 | 55 (33.1%) |
| 4 | 1 (0.6%) |
| ***Indication for surgery – no. (%)*** | |
| Colorectal cancer | 126 (75.9%) |
| Ulcerative colitis | 15 (9.0%) |
| Crohn's disease | 14 (8.4%) |
| Diverticular disease | 6 (3.6%) |
| Stricture | 3 (1.8%) |
| Benign polyp | 1 (0.6%) |
| Meckel’s diverticulum | 1 (0.6%) |
| ***Operation – no. (%)*** | |
| Anterior resection | 34 (20.5%) |
| Panproctocolectomy | 6 (3.6%) |
| Right hemicolectomy | 49 (29.5%) |
| Left hemicolectomy | 6 (3.6%) |
| Subtotal colectomy | 10 (6.0%) |
| AP resection | 15 (9.0%) |
| Small bowel resection | 4 (2.4%) |
| Ileocaecal resection | 8 (4.8%) |
| Proctectomy | 13 (7.8%) |
| Sigmoid colectomy | 7 (4.2%) |
| Transverse colectomy | 1 (0.6%) |
| Hartmann’s | 10 (6.0%) |
| Perineal reconstruction | 1 (0.6%) |
| Adhesiolysis | 2 (1.2%) |
| ***Surgical approach – no. (%)*** | |
| Open | 68 (41.0%) |
| Laparoscopic | 74 (44.6%) |
| Converted to open | 21 (12.7%) |
| Robotic | 3 (1.8%) |

**Supplementary Table 1.** Depicting overall demographics and clinical characteristics.

**Supplementary Table 2.** Fourteen instrumental Colorectal ERAS factors (ranging from day of operation to day 3 post-operation) assessed for compliance per patient displayed as a mean percentage compliance in pre-pandemic and peri-pandemic groups. Significance between the groups compared using Chi Squared analysis and represented as a p value.

|  | **Proportion of completion** | | |  |
| --- | --- | --- | --- | --- |
| **ERAS factor** | **Pre-pandemic  (n = 110)** | **Peri-pandemic**  **(n = 56)** | **Overall (n = 166)** | **p value** |
| ***Day 0 – no. (%)*** | | | | |
| Paracetamol as prescribed | 82 (74.5%) | 55 (98.2%) | 137 (82.5%) | **<0.0001** |
| Out of bed at all | 48 (43.6%) | 22 (39.5%) | 70 (42.2%) | 0.59 |
| Nutritional supplements >200 ml | 11 (10.0%) | 6 (10.7%) | 17 (10.2%) | 0.88 |
| Oral fluids >800 ml | 38 (34.5%) | 26 (46.4%) | 64 (38.6%) | 0.14 |
| ***Day 1 – no. (%)*** | | | | |
| Surgical Team r/v FBC + U&E | 106 (96.4%) | 55 (98.2%) | 161 (97%) | 0.51 |
| No intravenous fluids | 43 (39.1.%) | 29 (51.8%) | 72 (42.4%) | 0.12 |
| Nutritional supplements >400 ml | 24 (21.8%) | 15 (26.8%) | 39 (23.5%) | 0.48 |
| Normal food | 93 (84.5%) | 54 (96.4%) | 147 (88.6%) | **0.02** |
| Out of bed >6 h | 103 (93.6%) | 56 (100%) | 159 (95.8%) | 0.054 |
| ***Day 2 – no. (%)*** | | | | |
| Urinary catheter out | 59 (53.6%) | 41 (73.2%) | 100 (60.2%) | **0.02** |
| Epidural removed | 84 (76.4%) | 48 (85.7%) | 132 (79.5%) | 0.16 |
| ***Day 3 -no. (%)*** | | | | |
| CRP taken +/- senior review if >150 | 91 (82.7%) | 55 (98.2%) | 146 (88.0%) | **0.004** |
| Established oral intake | 96 (87.3%) | 55 (98.2%) | 151 (91.%) | **0.02** |
| Movicol OD since admission | 22 (21.8%) | 20 (30.8%) | 42 (25.3%) | 0.19 |

**Supplementary Table 3.** Post-operative complications rates in pre-pandemic and peri-pandemic Colorectal ERAS patients. Values are number (proportion). Significance between the groups compared using Chi Squared analysis and represented as a p value.

| **Complication** | **Pre-Pandemic (n = 110)** | **Peri-Pandemic**  **(n = 56)** | **Overall**  **(n = 166)** | **P-value** |
| --- | --- | --- | --- | --- |
| Any Complication | 53 (48.2%) | 23 (41.1%) | 76 (45.8%) | 0.39 |
| Wound Infection | 9 (8.2%) | 5 (8.9%) | 14 (8.4%) | 0.87 |
| Abdominal-Pelvic Collection | 8 (7.3%) | 1 (1.8%) | 9 (5.4%) | 0.14 |
| Post-operative nausea/vomiting | 42 (38.2%) | 17 (30.4%) | 59 (35.5%) | 0.32 |
| Ileus | 15 (13.6%) | 8 (14.3%) | 23 (13.9%) | 0.91 |
| Chest Infection | 3 (2.7%) | 5 (8.9%) | 8 (4.8%) | 0.08 |
| Myocardial Infarction | 2 (1.8%) | 0 (0%) | 2 (1.2%) | 0.31 |
| Deep Vein Thrombosis | 0 (0%) | 0 (0%) | 0 (0%) | N/A |
| Pulmonary Embolism | 0 (0%) | 0 (0%) | 0 (0%) | N/A |

**Supplementary Figures**

**Supplementary Figure 1.** Distribution of Colorectal ERAS pathway compliance in the peri-pandemic compared to pre-pandemic group.


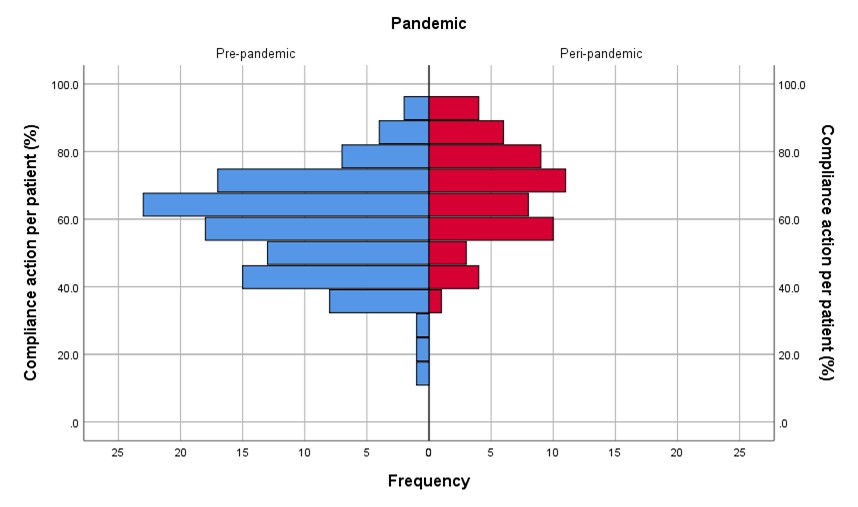


**Supplementary figure 2.** Distribution of length of stay in Colorectal ERAS pathway patients in the peri-pandemic and pre-pandemic groups.


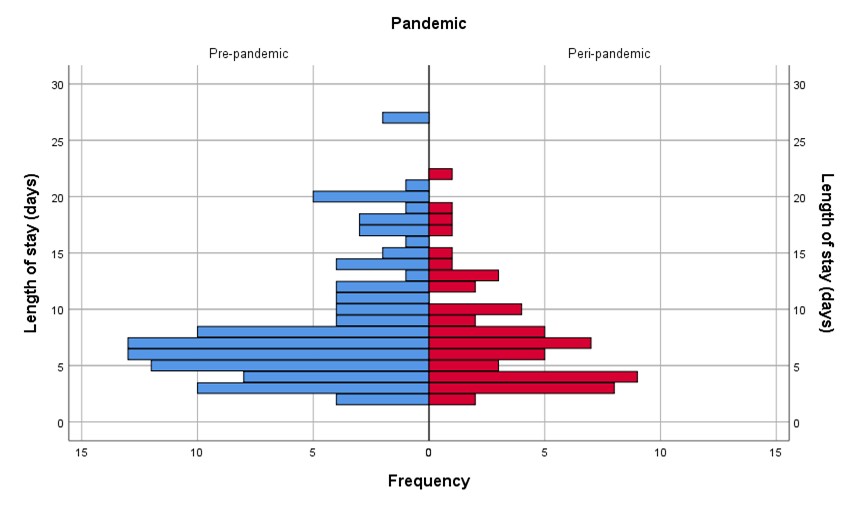

Supplement: znab208_Supplementary_Data [file znab208_Supplementary_Data.docx]
